# Electronic Structure of the Azide Group in 3′-Azido-3′-deoxythymidine (AZT) Compared to Small Azide Compounds

**DOI:** 10.3390/molecules14072656

**Published:** 2009-07-22

**Authors:** Fang-Fang Chen, Feng Wang

**Affiliations:** Centre for Molecular Simulation, Swinburne University of Technology, P. O. Box 218, Hawthorn, Melbourne, Victoria, 3122, Australia; Email: fchen@ict.swin.edu.au (F.C.)

**Keywords:** azide, azidothymidine, electronic structures, density functional theory

## Abstract

Theoretical calculations for some structural and electronic properties of the azide moiety in the nucleoside reverse transcriptase (RT) inhibitor 3′-Azido-3′-deoxythymidine (AZT) are reported. These properties, which include geometrical properties in three dimensional space, Hirshfeld charges, electrostatic potential (MEP), vibrational frequencies, and core and valence ionization spectra, are employed to study how the azide group is affected by the presence of a larger fragment. For this purpose, two small but important organic azides, hydrazoic acid and methyl azide, are also considered. The general features of *trans* Cs configuration for RNNN fragments [1] is distorted in the large AZT bio-molecule. Hirshfeld charge analysis shows charges are reallocated more evenly on azide when the donor group R is not a single atom. Infrared and photoelectron spectra reveal different aspects of the compounds. In conclusion, the electronic structural properties of the compounds depend on the specific property, the local structure and chemical environment of a species.

## 1. Introduction

Organic azides (N_3_-R) are considered flexible energy-rich functional molecules, which have been widely used in a number of areas such as material science, synthetic chemistry and biomedicine [2]. Apart from its potential applications in the development of explosive and superconducting materials [3,4] or as an intermediate in organic synthesis, the azide function also has important pharmaceutical applications as an enzyme inhibitor. For example, it has been used as a non-protonated inhibitor of human carbonic anhydrase [5]. Most azide-containing drugs are approved as active drug ingredients [2], and they are produced from synthesis rather than from natural products. The attachment of organic azides to nucleoside analogues through synthetic chemistry has opened a new avenue of anti-virus drug discovery. For example, the azide is an important functional group in an antiviral nucleoside analogue drug, 3’-azido-3’-deoxythymidine (AZT), which has been used as a successful reverse transcriptase (RT) inhibitor of human immunodeficiency virus (HIV). 

A number of theoretical and experimental studies using techniques like microwave spectroscopy (MW), X-ray and electron diffraction (ED) have been performed to study small covalently bonded azide compounds [6,7]. Thermal decomposition of some azide-containing compounds was studied through gas phase photoelectron spectroscopy [8,9]. X-ray and Ultraviolet electron spectroscopy studies have also revealed the electronic structures for azide ion and hydrazoic acid [10]. However, the majority of such theoretical efforts have focused on properties such as geometry, vibrational spectra and chemical bonding [1,2,11,12,13], and have been limited to either the isolated azide functional group or small azide-containing molecules. Little information is available on the responses of the azide group to the presence of other fragments in larger bio-molecule inhibitors, in comparison with small organic molecules. Since significant advances have been made for azide-containing drugs like AZT in anti-HIV therapies, more information regarding the behavior of the azide group and its interaction with fragments of varied size is needed and may hold the key for new drug discovery.

AZT is a pyrimidine nucleoside analogue with an azide replacing the hydroxyl group at the 3’ position on the sugar ring of thymidine. The role is to terminate duplication of virus RNA. Despite its successful clinical applications, AZT suffers from long term resistance and toxicity problems. For drugs and drug candidates, their bio-activities, functions and interactions with the environment are closely related to their electronic structures. Although a number of studies by various methods and techniques like X-ray crystallography and theoretical calculations have been employed to investigate the structures and their interactions, lots of information is still unknown or unclear. For example, what’s the role of azide in AZT and its intramolecular interaction with the thymine and sugar moieties in AZT? In this study, electronic structural calculations of AZT are carried out, in comparison with simple organic azide compounds such as hydrazoic acid (HN_3_) and methyl azide (CH_3_N_3_), in order to reveal potential structural changes in the azide group when interacting with fragments of varied sizes. 

## 2. Results and Discussion

### 2.1. Structures and Hirshfeld Charge Distributions

Optimized geometries of AZT, HN_3_ and CH_3_N_3_, focusing on the azide fragment, are reported in Table 1. Results from the present study are compared with other theoretical means as well as available experiments. Excellent agreement has been achieved, indicating that the B3LYP/6-311G** model used in the present study is able to accurately predict the structures of the various species. The present results reproduce general geometric features found by earlier studies [1,12], i.e. (i) a *trans* Cs configuration preference for the RNNN fragment (except for AZT); (ii) a slightly bent N-N-N unit; and (iii) distinctly different bond lengths between N_(1)_-N_(2)_ and N_(2)_-N_(3)_. In general, geometries of the azide group produced through different methods don’t change significantly in different chemical environment across the species. For example, variations of the relevant bond lengths and bond angles are negligible, as shown in Table 1. 

**Table 1:** Responses of selected structural parameters of azide in AZT, HN_3_ and CH_3_N_3._

Structure parameters	AZT			HN_3_			CH_3_N_3_		
	This Work	BLYP/6-31+G (d, p) [14]	Expt. [15]	This Work	MP2/TZ2P [13]	Expt. [13]	This Work	B3LYP/6-311++G** [16]	Expt. MW [17]
N_(1)_-N_(2)_(Å)	1.23	1.25	1.25	1.24	1.24	1.24	1.23	1.23	1.24
N_(2)_-N_(3)_(Å)	1.13	1.16	1.12	1.13	1.14	1.13	1.14	1.14	1.13
R- N_(1)_ (Å)	1.48	1.49	1.49	1.02	1.02	1.02	1.47	1.47	1.46
∠N_(1)_-N_(2)_-N_(3)_(°)	173.6	171.6	173.2	171.5	171.6	171.3	173.2	173.3	
∠R-N_(1)_-N_(2)_(°)	115.9	116.8	115.0	110.1	109.5	108.8	116.1	116.1	117.0
∠R-N_(1)_-N_(2)_ –N_(3)_ (°)	-177.4			180.0			180.0	180.0	

Although the bond lengths between N_(1)_-N_(2)_ and N_(2)_-N_(3)_ of the azide moiety are distinctly different within a given compound, the corresponding azide bond lengths are almost identical across the three species. For example, the N_(1)_-N_(2)_ bond length of the three species lies in the vicinity of 1.23 Å, which is almost the same as the bond length of 1.24 Å in an isolated N=N double bond [18]. A triple bond character is observed for the terminal N_(2)_-N_(3)_ bond with a bond length of 1.13 Å, compared to an experimental value of 1.10 Å [19]. Some studies suggest that azide has an apparently pentavalent central nitrogen atom [12,20], in which the structure of the N-N-N chain can be given by (1) [20,21]:

*N* = *N* ≡ *N* (1)

The azide bond angle, ∠N_(1)_-N_(2)_-N_(3)_, is 173.6°, 173.2° and 171.5° in AZT, CH_3_N_3_ and HN_3_, respectively, which clearly indicates a non-linear asymmetric chain structure. With regards to point group symmetry, HN_3_ and CH_3_N_3_ both belong to the C_s_ group with a dihedral R-N-N-N bond angle of 180°, while in AZT this dihedral angle, i.e., C_(3’)_-N-N-N, becomes -177.4° as it is appended to a large unsymmetrical thymidine fragment.

**Figure 1:**
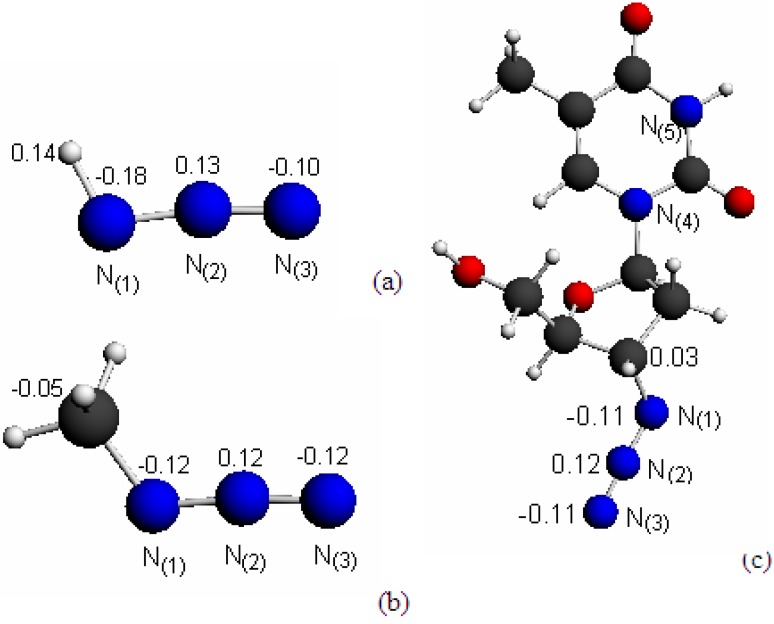
Structures and Hirshfeld charges of: (a) hydrazoic acid (HN3), (b) methyl azide (CH3N3) and (c) 3′-Azido-3′-deoxythymidine (AZT).

The Hirshfeld charges (Q^H^) of nitrogen atoms, which were calculated based on the LB94/et-pVQZ model, are marked on the azide in the three structures given in Figure 1. The major similarity in Hirshfeld charges among the azide moieties is that the middle N_(2)_ atom has positive charges whereas the terminal N_(1)_ and N_(3)_ atoms have negative charges, although the Hirshfeld charge distribution pattern is quite different in the various species. When R is a single H atom in HN_3_, a typical electron donor, the Q^H^ on H has an accumulated charge of 0.14 a.u., which directly attracts negative charges on N_(1)_ atom to -0.18 a.u. [Figure 1 (a)], whereas the terminal N_(3)_ of the same species is less negative with Q^H^ of -0.10 a.u.. When R≠H, the Hirshfeld charge on the carbon atom in fragment R, which directly connects with N_(1)_, is very small and almost neutral. For example, the Q^H^ of this carbon is 0.03 a.u. and -0.05 a.u. in AZT and CH_3_N_3_, respectively. As a result, the terminal Ns of the azide groups in CH_3_N_3_ and AZT exhibit certain symmetrical distribution in Q^H^ on both N_(1)_ and N_(3)._ Both terminal Ns of azide are approximately -0.12 a.u. in CH_3_N_3_ and -0.11 a.u. in AZT. 

The opposite signs of the Hirshfeld charge on those atoms connecting directly to azide suggest different roles for the R fragments in the species. Combined with Q^H^, the structure is presented as:

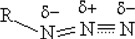
(2)


**Figure 2:**
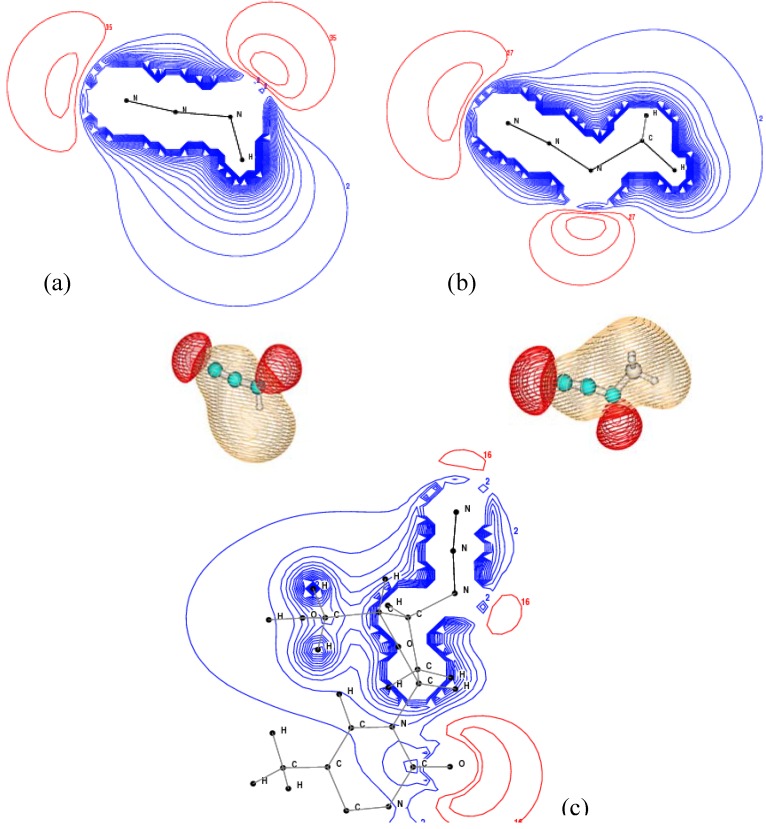
Molecular electrostatic potentials (MEPs) of (a) HN_3_, (b) CH_3_N_3_, and (c) AZT.

This azide bonding structure proposed by Equation (2) contains certain similarities to the structure II proposed by Klapötke [12,20] from inorganic azides. The spatial arrangement of molecules is reflected directly by their electron distributions. The bonding structure proposed in Equation (2), is supported by the molecular electrostatic potentials (MEPs) of the species presented in Figure 2. 

### 2.2. IR Spectroscopy

Azide vibrational motions could easily be identified from the infrared (IR) spectra of the three species, which display minor changes reflecting their specific molecular environments. Four characteristic azide vibrational frequencies, including two stretch modes and a pair of bend modes, are listed in Table 2. The spectral peaks in the vicinity of 2,250 cm^-1^ (ν_1_) and 1,330 cm^-1^ (ν_2_) are assigned to the asymmetric and symmetric NNN stretches, respectively. The other two bands are assigned to the bend and torsion modes. The results in the present study are in good agreement with the experimental data with regards to the bend modes but less accurate with respect to the stretch modes without any scaling. For example, the predicted bend modes, with the exception of the NNN bend mode of CH3N3, are within 5 cm^-1^ of the experimental values. The agreement with experimental data for the asymmetric NNN stretch, which are available for all species, is very good when a scale factor of approximately 0.94 is applied. It is seen in the Table 2 that vibration frequencies of methyl azide and AZT are closer in wavenumber than those of HN_3_ due to their reduced masses. The torsion (ν_3_) and bend (ν_4_) modes are reversed in HN_3,_ compared to AZT and CH_3_N_3_, reflecting the size of the fragments attached to azide. In HN_3,_ the small and light H atom leads to an easier torsional motion than bending motion so that v3 > v4. However, the R fragments in AZT and CH_3_N_3_ significantly change the inertia and lead to an opposite trend of v3 < v4, that is, bending is easier than torsional motion in CH_3_N_3_ and AZT. 

**Table 2:** Infrared (IR) spectra of azide in HN_3_, CH3N3 and AZT (in cm^-1^).

HN_3_			CH_3_N_3_			AZT			
This Work ^a^	B3LYP/6-31G** [22]	Expt. [23]	This Work*	B3LYP/6-31G** [22]	Expt. [23]	This Work ^a^	BLYP/DZVP [24]	Expt. [25]	Assigned Vibration Mode
2278	2294	2129	2247	2264	2106	2242	2103	2102	ν_1_, Asym. NNN str.
1300	1313	1264	1343	1474	1272	1328			ν_2_, Symm. NNN str.
533	529	537	666	665	666	658			ν_4_, NNN torsion
603	603	606	574	574	560	572			ν_3_, RNNN bend

^a^ B3LYP/6-311G** model.

### 2.3. Core Shell Photoelectron Spectroscopy

Core shell ionization spectra (IP) reveal subtle differences in the chemical environments of atoms in a molecule. Vertical ionization energies for the N1s sites of the three species, based on the “meta-Koopman” theorem [26] are given in Table 3. Three out of five N1s sites in AZT belong to the azide moiety, whereas N_(4)_ and N_(5)_ are components of the thymine (the nucleic base) moiety. 

**Table 3:** Vertical ionization energies of N1s sites in HN_3_, CH_3_N_3_ and AZT in the inner-shell (eV).

N1s sites	AZT	CH_3_N_3_	HN_3_ (This work)	HN_3_ (Expt [10])
N_(1)_	405.23	404.87	405.05	399.5
N_(2)_	408.07	407.63	408.10	403.6
N_(3)_	405.62	405.16	405.57	400.4
N_(5)_ (base)	404.73			
N_(4)_ (base)	405.18			

The IP results of NH_3_ are consistent with the experimental data, in particular the site specific energy trend and energy splitting. Chemical shifts between N_(2)_ and N_(3)_ and between N_(3)_ and N_(1)_ given by our LB94/et-pVQZ model are 2.53 eV and 0.52 eV, respectively, which are slightly smaller than the HN_3_ experimental observation of 3.2 eV and 0.9 eV [10]. Given that the present calculations are simple and involve a number of approximations, the calculation results are attractive. 

The N1s binding energy spectra of the three species exhibit certain common properties. The most noticeable feature is the large energy gaps of over 2.5 eV between the middle nitrogen atom N_(2)_ and the remaining nitrogen atoms N_(3)_. Although a global chemical shift in the three spectra is observed, the energy gaps between N_(2)_ and N_(3)_ are in the vicinity of 2.50 eV, with a slight increase as the size of the molecule decreases. For example, the energy gap is 2.45 eV, 2.47 eV and 2.53 eV for AZT, CH_3_N_3_ and HN_3,_ respectively. The energy ordering of the azide N1s is consistently given by N_(1)_<N_(3)_<N_(2)_ for all species, which is in accordance with the Hirshfeld charge distribution (see Figure 2). The terminal N atoms with negative charges are associated with lower ionization energies, while positive charge on the central atom has a larger IP, indicating that the N_(2)_ site lies in a very different chemical environment from their terminal N atoms. Small chemical shifts (0.3 to 0.5 eV) are observed between N_(1)_ and N_(3)_, depending on the R fragments.

**Figure 3:**
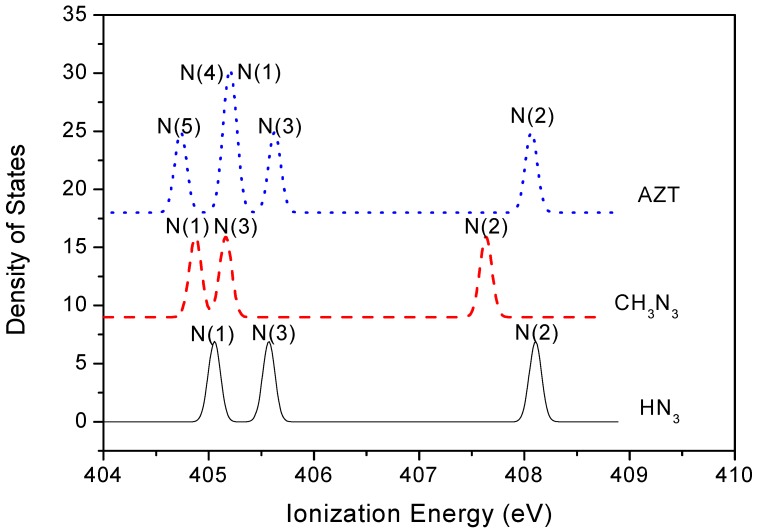
The simulated N-K binding energy spectra of AZT, CH_3_N_3_ and HN_3_.

The size of the fragments does not seem to correlate directly to chemical shift. Figure 3 gives the vertical core ionization spectra of AZT, methyl azide and hydrazoic acid. In the spectral simulation, full width at half maximum (FWHM) was 0.13 eV, which is a much higher resolution than currently available with synchrotron sourced x-ray photoemission spectroscopy, with a typical resolution of approximately 0.40 eV for N-K spectra [27]. The N1s spectra of AZT (apart from the thymine N sites) exhibit a larger degree of similarity to the N1s spectra of hydrazoic acid than to methyl azide. For example, the chemical shift between AZT and HN_3_ on N_(2)_ is -0.03 eV and on N_(3)_ is only 0.05 eV according to our results. The corresponding shift between CH_3_N_3_ and HN_3_ is -0.47 eV on N_(2)_ and -0.41 eV on N_(3)._ The prominent global red shifts found in CH_3_N_3_ may be caused by an inductive effect from the methyl group. 

The N1s spectrum of AZT is the result of interactions dominated by five N1s sites, which may also affect the spectral peak positions of the azide N1s sites. It is possible that the interactions among the five N1s sites and the large AZT fragment result in an energy cancellation, leading to certain similarities in the N-K spectrum between AZT and hydrazoic acid. It is also noted that an accidental energy “degeneracy” exists in AZT, that is, the N_(1)_ site in the azide group and the N_(4)_ site in the thymine base moiety, with an energy splitting of only 0.05 eV. 

### 2.4. Valence Shell Photoelectron Spectroscopy

Vertical ionization energies of the valence shell of the compounds were produced using the DFT based SAOP/et-pVQZ model and Green’s function (OVGF/TZVP model) calculations. The results are listed in Table 4, together with available experimental IP results. A total of seven valence orbitals in each species are dominated by azide. Those azide orbitals are labeled according to the SAOP/et-pVQZ calculations. This SAOP/et-pVQZ model has been proven to be an effective method to describe the IPs in the entire valence shell [28], whereas OVGF provides information only in the outer valence space. However, OVGF provides accurate first IPs for many biomolecules and SAOP is usually unable to provide accurate ionization energies for outermost orbitals, especially the first IP. The combination of SAOP and OVGF calculations complement each other. It is seen that the agreement between SAOP and OVGF is very good when the orbital is not the outermost orbital, such as orbitals 8a′, 1a″ and 7a′ in HN_3_ and 10a′, 9a′ in CH_3_N_3._

**Table 4:** Vertical ionization energies of AZT, CH_3_N_3_ and HN_3_, dominated by azide, using SAOP/et-pVQZ and OVGF/TZVP models (eV).

Orbitals	SAOP/et-pVQZ	OVGF/TZVP	Expt. [10]
[HN_3_]			
2a″	11.51	10.13 (0.91)	10.7
9a′	12.75	11.87 (0.92)	12.2
8a′	15.76	15.88 (0.88)	15.5
1a″	17.34	17.04^*^ (0.81)	16.7
7a′	17.35	17.17^*^ (0.86)	17.4
5a′	29.16		
4a′	33.47		
[CH_3_N_3_]			
3a″	10.87	9.35 (0.91)	
12a′	12.04	10.96 (0.92)	
10a′	15.58	15.54 (0.88)	
9a′	17.28	17.26 (0.85)	
1a″	17.32		
6a′	29.05		
5a′	33.18		
[AZT]			
69a	10.78		
63a	12.34		
47a	15.98		
43a	17.23		
42a	17.43		
25a	29.36		
20a	33.48		

^*^ Orbital ordering of experiment has 1a” and 7a’ swap comparing to this work, an *ab initio*-SCF molecular orbital (MO) calculations was used to give the ordering in experiment.

**Figure 4:**
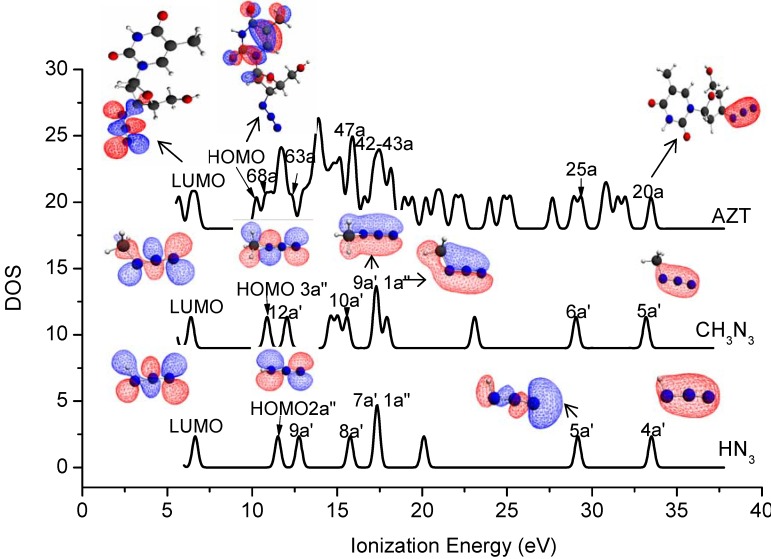
Simulated valence binding energy spectra (FWHM=0.40 eV) of AZT, CH_3_N_3_ and HN_3_, based on SAOP/et-pVQZ calculations. The azide dominant orbitals are labeled in the spectra with selected orbitals contour plots.

Vertical valence ionization spectra of AZT, HN_3_ and CH_3_N_3_ were simulated using the SAOP/et-pVQZ model, i.e., the results given in Figure 4. A Gaussian broadening function is used with an FWHM of 0.4 eV in the simulation. Although valence orbitals are largely delocalized, it is still able to identify orbitals which are dominated by a functional group [29]. The orbital contributions which are dominated by azide in each compound are labeled in the spectra. One of the most significant features among the azide compounds is that all the lowest unoccupied molecular orbitals (LUMOs) are dominated by azide, whereas only the highest occupied molecular orbitals (HOMOs) of HN_3_ and CH_3_N_3_ are dominated by azide, that is, the HOMO of AZT does not locate on the azide moiety. The inner valence space (i.e., IP > 25 eV) exhibits an apparently clear pattern in the spectra, due to the large energy splitting in this region. For example, orbitals 4a′ (HN_3_), 5a′ (CH_3_N_3_) and 20a (AZT) that are positioned at ca. 33 eV possess σ bond characters, which are formed through overlap of 2s orbitals of three nitrogen atoms. Orbitals 7a′ and 1a″ in HN_3_ (it should be noted that these orbitals were labeled as 6a′ and 1a″ in a previous INDO calculation [10]), orbitals 9a′ and 1a″ in CH_3_N_3_ as well as orbitals 42a and 43a in AZT are clearly the π orbitals (only orbitals 9a’ and 1a” in CH_3_N_3_ are given in as insert in Figure 4). 

## 3. Theoretical Methods and Computational Details

The chemical structure of 3’-azido-3’-deoxythymidine (zidovudine, i.e. AZT,) is displayed in Figure 1, together with the structures of hydrazoic acid (HN_3_) and methyl azide (CH_3_N_3_). Only the azide (N_3_) nitrogen atoms are labeled in the figure. A density functional theory (DFT) based B3LYP/6-311G** model was employed to obtain geometries and infrared (IR) spectra of the species. The optimized structures are real minimum structures as they are not associated with any imaginary frequencies. Further single point calculations using DFT models such as LB94/et-pVQZ [30] and statistically averaged orbital potential (SAOP) model SAOP/et-pVQZ model [31] are employed to explore properties of the species. Here the et-pVQZ is an even-tempered polarized valence quadruple-zeta Slater type basis set [32]. Core events, such as atomic charges according to the Hirshfeld scheme [33] and vertical core ionization potentials (IPs) are produced using the LB94/et-pVQZ model. It has been proven that the LB94/et-pVQZ model produces core IPs of other nucleosides such as cytidine [34] with a competitive quality from a recently developed CV-B3LYP model [35]. The SAOP/et-pVQZ model has been proven to be accurate and capable of producing valence ionization potentials (vertical) up to inner valence shell, i.e., up to an energy range of approximately 25 eV [29,36,37]. Computational chemistry packages such as Gaussian 03 [38] and Amsterdam Density Function (ADF) [39,40] are employed in the quantum mechanical calculations and spectral simulations. ADFView [39,40] and Molden [41] are employed for visualization of the structures and properties.

## 4. Conclusions

Electronic structural responses of azide in 3’-azido-3’-deoxythymidine have been studied using quantum mechanical density functional theory models, and compared to the properties of small azide compounds such as hydrazoic acid and methyl azide. A number of properties, such as geometrical structures in three-dimensional space, atomic charge distributions according to the Hirshfeld scheme, molecular electrostatic potential, ionization energies in both core and valence spaces, as well as binding energy spectra are analyzed and compared across the compounds. It is found that the fragments (R) produce small perturbations to the geometry of the azide moiety, but they apparently alter atomic charge distributions, as reflected by their Hirshfeld charges and molecular electrostatic potentials. The Hirshfeld charges show that methyl azide and AZT exhibit similar symmetric charge distribution patterns. Similarities between methyl azide and AZT are also found in their infrared spectra, as their bend modes exhibit lower frequencies than their torsion modes, which is in contrast to the azide bend and torsion frequencies in hydrazoic acid. While the inner-shell azide ionization spectra displays certain analogous in the patterns between hydrazoic acid and 3’-azido-3’-deoxythymidine, their valence binding energy spectra indeed show similarities in hydrazoic acid and methyl azide. In conclusion, the properties of azide do not indicate certain size dependent trends with the fragments in hydrazoic acid, methyl azide and 3’-azido-3’-deoxythymidine. Rather, the properties depend on a specific property, the local structure and chemical environment of the species.
